# Azotobacter vinelandii gene fitness following carbon shift from sucrose to acetate, succinate and glycerol

**DOI:** 10.1099/mic.0.001643

**Published:** 2026-01-16

**Authors:** Carolann M. Knutson, Brett M. Barney

**Affiliations:** 1 Department of Bioproducts and Biosystems Engineering, University of Minnesota, St. Paul, MN 55108, USA; 2 Biotechnology Institute, University of Minnesota, St. Paul, MN 55108, USA

**Keywords:** acetate, *Azotobacter*, carbon metabolism, glycerol, succinate, Tn-Seq

## Abstract

Nitrogen-fixing microbes are a primary contributor of this important nutrient to the global nitrogen cycle. Biological nitrogen fixation (BNF) through the enzyme nitrogenase requires extensive energy that in whole cells is generally studied during the oxidation of carbohydrates such as sugars. The nitrogen-fixing bacterium *Azotobacter vinelandii* is a model diazotroph for the study of aerobic BNF. Much is known about metabolism in *A. vinelandii* when cultured on a simple medium where energy is provided primarily in the form of sucrose or glucose. Outside of the laboratory, this soil bacterium grows on metabolites primarily derived from plant root exudates or from the degradation of dead plant matter. In this work, we expand on previous studies looking at genes that are essential to BNF in *A. vinelandii* when grown on sucrose medium using transposon sequencing (Tn-seq). We applied Tn-seq to determine the genes essential to growth when the medium was shifted to acetate, succinate or glycerol as the primary carbon and energy source to fuel both growth and BNF. A global overview of the genes of central metabolism and those directing substrates toward central metabolism, along with a selection of unexpected genes that were essential for specific growth substrates, is provided.

## Data Availability

Raw sequencing reads are available on the National Center for Biotechnology Information (NCBI) Sequencing Read Archive under BioProject ID PRJNA701611, accession numbers SRX31273225, SRX31273455, SRX31273519, SRX31273520, SRX31273521 and SRX31273522.

## Introduction

*Azotobacter vinelandii* is a model microbe for the study of aerobic biological nitrogen fixation (BNF). In laboratory experiments, *A. vinelandii* is typically grown on Burk’s medium containing sucrose as the sole carbon source [1,2]. Sucrose, a disaccharide of glucose and fructose, is found in the soil provided by plants through either root exudate or by decomposition [3]. While sugars are the most abundant organic carbon source in soil, carboxylic and amino acids are also found [3]. Interestingly, *A. vinelandii* prefers certain carboxylic acids to sugars in a process referred to as organic acid-mediated carbon catabolite repression (OAM-CCR) to differentiate it from CCR, in which sugars are preferentially consumed [4]. For example, diauxic growth of *A. vinelandii* occurs when grown on a mixture of acetate and glucose, where acetate is consumed first [1,5, 6]. Other organic acids that inhibit glucose uptake in *A. vinelandii* include citrate, isocitrate and 2-oxoglutarate, while pyruvate, oxaloacetate and succinate exhibited lesser degrees of inhibition [1].

Growing microbes on different carbon compounds alters the bioenergetics and flux through central metabolism [7], thus changing the concentration of cellular metabolites. Additionally, since metabolic pathways are the main providers of NADH and NADPH [8], evaluation of these pathways is critical to understanding how carbon sources impact energetically expensive cellular processes like BNF. For example, when grown on acetate, *A. vinelandii* had lower levels of nitrogen fixation than when grown on glucose, though these lower levels of nitrogen fixation appeared to support faster growth [1,6]. Likewise, in mutants engineered to release excess ammonium, carbon-dependent excretion was shown in which sucrose, glucose and glycerol supported comparable ammonium production while succinate, fumarate, malate, pyruvate and acetate showed severe reductions (20–40% of the ammonium produced with sugars) [9]. One explanation for the differences in the ammonium production has been varying levels of 2-oxoglutarate [9]. 2-Oxoglutarate binds to the *nif* transcriptional activator, NifA, to signal the carbon status of the cell and promote transcription of *nif* genes [10,11]. Transcription of *nif* genes does not occur when no 2-oxoglutarate is bound to NifA. Thus, carbon sources must provide sufficient levels of 2-oxoglutarate to support active nitrogen fixation.

As such, the carbon source and its effect on BNF cannot be ignored in the design of biofertilizer strains. Many *A. vinelandii* engineered strains have reported results for ammonium production only when the engineered strain was grown on sucrose [12,15]. If the end goal is to provide ammonium to plants in a typical soil environment, these strains need to be re-evaluated with a range of carbon sources more representative of the final growth conditions. Understanding the performance of strains in varied environments will help determine when engineered mutants may fail in practical applications where carbon source mixtures are present, as growth on organic acids (a potentially preferred carbon source) will likely not provide the ammonium predicted by sucrose-only laboratory growths. This suggests that in engineering biofertilizer strains, we should evaluate strains under more conditions and substrates, similar to other strain engineering practices, as the carbon source or the presence of OAM-CCR can have consequences on the ammonium yields.

Global studies such as fluxomics and RNA-seq have identified upregulated pathways and genes in *A. vinelandii* [16,18] and its close relative *Pseudomonas aeruginosa* [7] on certain carbon sources. While these studies are useful, there is still a lack of information for many other physiologically relevant carbon sources. In this study, we used transposon sequencing (Tn-seq) to identify genes important for growth on acetate, succinate and glycerol. We chose readily available carbon sources, and they also entered central metabolism at different points. The benefit of using Tn-seq over RNA-seq is that we can probe genes whose expression may not fluctuate in response to the carbon source, such as transcription factors. This study complements our previous publication [19], where we examined the fitness of *A. vinelandii* under diazotrophic and non-diazotrophic growth on sucrose. The data described in this study were produced simultaneously with the data presented in our previous study and can be directly compared. Collectively, these studies provide a database that researchers can use to inform future research targets and provide a better picture of how this strain deals with changing bioenergetics when provided with alternative substrates.

## Methods

### Strains, media and growth conditions

*A. vinelandii* DJ (ATCC BAA-1303) was grown aerobically in modified Burk’s (B) medium (20 g l^−1^ sucrose, 0.2 g l^−1^ MgSO_4_·7H_2_O, 90 mg l^−1^ CaCl_2_·2H_2_O, 0.25 mg l^−1^ Na_2_MoO_4_·2H_2_O, 5.0 mg l^−1^ FeSO_4_·7H_2_O and 10 ml l^−1^ 100X phosphate buffer composed of 20 g l^−1^ KH_2_PO_4_ and 80 g l^−1^ K_2_HPO_4_) [2]. B media were supplemented with one of the following carbon sources to replace sucrose at 20 g l^−1^: sodium acetate, sodium succinate or glycerol. Where indicated, nitrogen was supplied as urea (10 mM). Cultures were grown as 250 ml volumes in 500-ml Erlenmeyer shake flasks at 30 °C and 180 r.p.m.

### Tn-seq samples

Tn-seq samples were prepared concurrently with the samples described in a previous publication [19]. At the time when these experiments were initiated, two replicates were an acceptable experimental design for Tn-Seq studies. The inclusion of four different substrates means that our results generated eight data points (duplicates for each) to compare and contrast results with one another. Samples were sequenced at the University of Minnesota Genomics Center, as previously described using a custom method [20]. Similarly, all samples were subjected to the same processing and analysis pipeline. As all samples were initially isolated onto B media with urea, library statistics and essential genes are the same as previously presented [19]. Samples were harvested after approximately seven generations of growth. OD_600_ values at the time of harvest are shown in Table 1. The seventh-generation target was selected to provide a sufficient number of doublings to differentiate subtle as well as pronounced variations. Fitness of non-essential genes was calculated using the following equation [21], where $N_{i,t_{1}}$ and $N_{i,t_{2}}$ were the proportion of the gene before and after growth in the selective conditions and *d* is the expansion factor calculated as the OD_600,t2_ /OD_600,t1_.

**Table 1.** Cell density and mapped reads

Sample	OD_600_	Expansion factor	Mapped reads
Acetate #1	6.06	121.1	15,001,388
Acetate #2	4.94	98.7	16,871,019
Succinate #1	6.00	120.0	17,650,273
Succinate #2	7.70	154.1	15,571,966
Glycerol #1	6.75	135.0	18,910,633
Glycerol #2	6.77	135.5	16,842,781


$$W_{i}=\;\frac{ln\left(N_{i,\;t_{2}}\;\times \;\;\frac{d}{N_{i,\;t_{1}}}\right)}{ln\left(\left(1-N_{i,\;t_{2}}\right)\;\times \;\frac{d}{1-N_{i,\;t_{1}}}\right)}$$

### Software

Visualization was done in R (R Core Team 2020) using the tidyverse [22], gggenes [23], patchwork [24] and ggtext [25] packages.

### Code availability

All code is available on GitHub at https://github.com/Brett-Barney-UMN/CK-Tn-Seq for the Tn-seq analysis. Code for making figures and other intermediate data analysis can be provided upon request.

### Genetic constructs

To validate the results of specific findings related to glycerol metabolism, we constructed two plasmids to target specific gene disruptions. We cloned the *spuA* gene and flanking regions with the primers 5′NNN**GAATTC**A GACAGTCAGC TCCAGCTCCT CGAAGC3′ and 5′NNN**AAGCTT**C AGGTGCAGCG CTTCGCCGTG ACGGAAG3′ into the pUC19 derivative pBB284 [26]. We then used primers 5′NNN**GGATCC**T AGCGAGCGAA TTGCAAGGAG TGTCATC3′ and 5′NNN**GGATCC**C ATGGCATCAT CCTGACTGTC GG3′ to remove the *spuA* gene. Finally, we incorporated a tetracycline cassette from pBBTET6 to complete the new plasmid pSPUA3 [27]. We cloned the *potHI* genes and flanking regions with the primers 5′NNN**GAATTC**C TGCGGCAAGT CGACCCTGCT GCGCAT3′ and 5′NNN**AAGCTT**C TCACCGACTT CCAGTCGATC GCCTG3′ into pBB284. We then digested the resulting plasmid with BglII to remove a large segment of *potHI* (~1400 bp) and incorporated a tetracycline cassette from pBBTET6 to complete the new plasmid pPOTGHI. The completed plasmids were used to transform *A. vinelandii* using methods previously described [28]. Completed strains AZBB760 and AZBB763 were confirmed using the primers 5′CTGCAGGCAG TCGACGTACT CGTCGAGGTC GTC3′ and 5′GATATGGTTG AGGAACAGCT CGCTCATG3′ for pSPUA3 and 5′CTCCTGTTCT TGAGTGGAG TTGAGATCAT G3′ and 5′GCGTGCTGGC CGAGACCATG ATCTC3′ for pPOTGHI. All plasmid sequences are available upon request.

## Results and discussion

### Library statistics

The goal of this study was to identify differences in the metabolism of *A. vinelandii* between sucrose and the alternative carbon sources acetate, succinate and glycerol during diazotrophic growth. The initial transposon mutant library for all samples was isolated on standard Burk’s (B) media with sucrose as the sole carbon source and urea as the nitrogen source. Isolation with a nitrogen source ensured that mutants with insertions in genes important to nitrogen fixation were retained while building the transposon mutant library. The transposon mutant library and, therefore, the mapped reads for the t1 samples for this data were previously presented [19]. This same library was used to inoculate the samples described here, and all samples were tested as duplicates. These samples were subjected to a carbon shift by inoculating the mutants pre-grown on sucrose and urea to either acetate, succinate or glycerol. The t2 mapped reads for the new samples described in this work (acetate, succinate and glycerol) are presented in Table 1. The minimum number of mapped reads for any one sample was 15,001,377, while the max was 18,910,633. The average number of mapped reads for all six new samples was 16,808,010.

Because our library was prepared on B sucrose supplemented with urea, we can only determine the fitness of genes that were non-essential for non-diazotrophic sucrose growth. We selected this carbon shift approach due to cost and time constraints and theorized that many genes important to growth on the alternative carbon sources would be retained, providing a favourable compromise to determine fitness. Also, due to sample limitations, we chose to only study diazotrophic growths (no added nitrogen), as we were most interested in metabolism differences in the context of nitrogen fixation. One benefit of this experimental design is that by isolating mutants on sucrose (or any carbon source different from the challenge condition), we were able to measure fitness for essential genes. Similar to our previous analysis, we only included genes that had an average greater than 50 counts in the t1 library and genes for which none of the samples contained a zero value. While genes with a zero value (i.e. no reads were detected in the t2 sample) still provide information, we cannot be certain when in the ∼seven generations the gene disappeared from the population. Essentially, zero values represent categorical data in a numerical data set. Fitness values for all genes are provided in File S1 (available in the online Supplementary Material).

To provide a global view of the changes seen in the data set, we chose to focus on those genes whose loss did not affect growth on sucrose (W ∼ 1). Since fitness differences may be a result of differential flux through a pathway, we wanted to find genes that seemed specific to a certain carbon source by looking at those genes that did not seem important to sucrose. Removing genes with zero values in any of the five conditions left 3,705 genes with which we could compare. Of these, 2,825 had a fitness between 0.9 and 1.1 on sucrose, and 2,807 had a distance between replicates less than 0.2. When we compared only genes with non-zero and tight values from the three alternative carbon sources to sucrose, 424 genes showed a difference in fitness greater than 0.2 fitness units. These lists were further broken down by the media where that difference was observed. In addition to genes that differed by 0.2 units, we also calculated those genes that differed by 0.3 and 0.4 units. Only 85 genes differed from sucrose by more than 0.4 units, of which 55 were specific to growth on acetate.

### EMP, ED and PP pathways

**Fig. 1.**
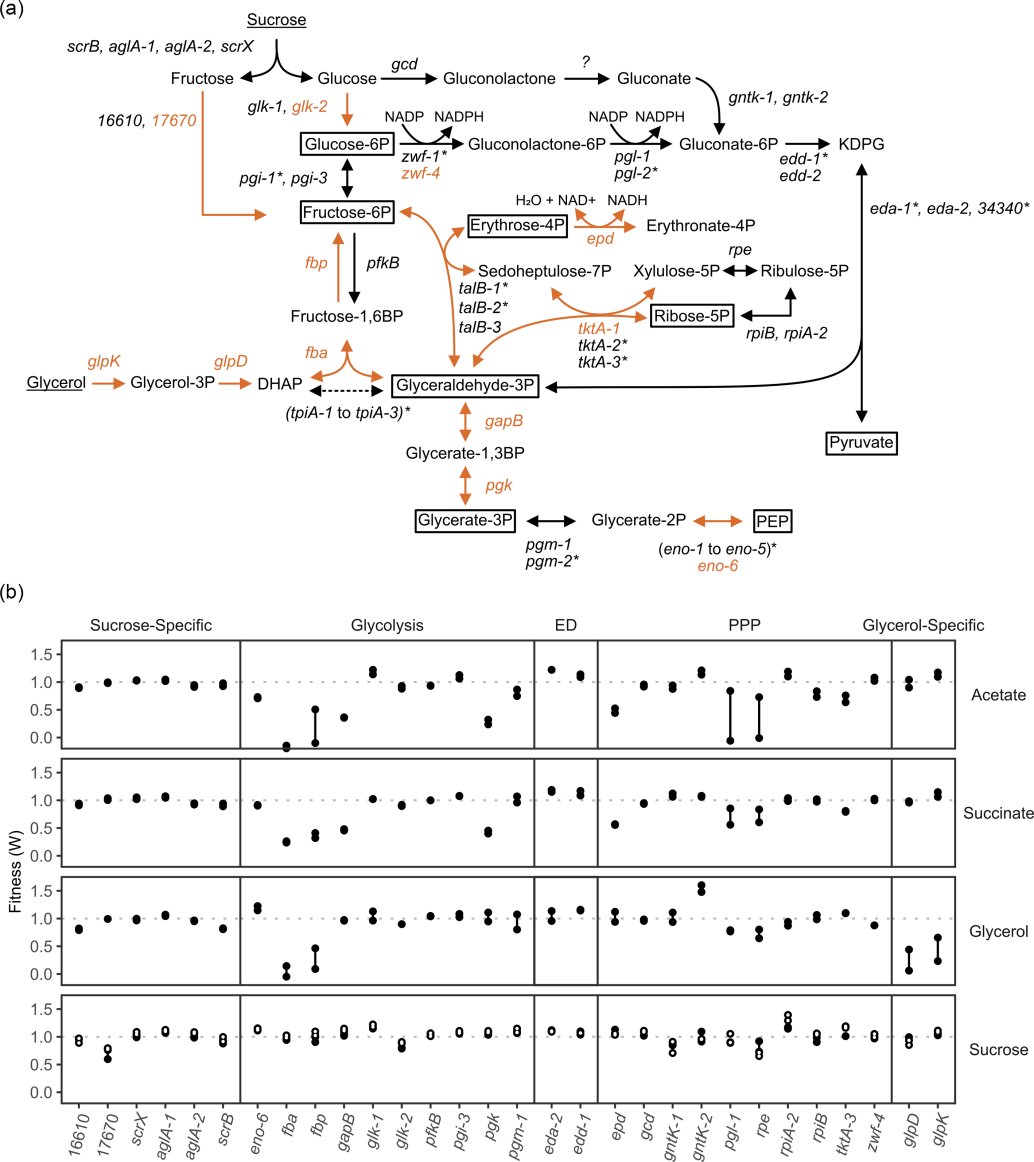
Putative enzymes involved in glycolysis, ED and PP pathways in *A. vinelandii* as determined by the KEGG database. (**a**) Carbon sources used in this study are underlined. Dotted lines indicate that no data were obtained for that reaction. Orange lines indicate that a difference was seen between carbon sources. Boxed intermediates are the essential metabolic precursors. Genes with an asterisk had low counts in the t_1_ samples, indicating they may be essential to growth under the conditions (sucrose and urea) in which the library was isolated. (**b**) Fitness of genes shown in panel (a). Closed circles were grown without nitrogen, while open circles were grown with nitrogen (urea). Numbers represent Avin_# designations for those genes which are not named.

To investigate the effects of different carbon sources on enzymes in the Entner–Doudoroff (ED), pentose phosphate (PP) and Embden–Meyerhof–Parnas pathways, we assembled genes belonging to these pathways using the Kyoto Encyclopedia of Genes and Genomes (KEGG) database for *A. vinelandii* DJ (Fig. 1). In addition to these pathways, we also included genes required to break sucrose down into glucose and fructose. Though *A. vinelandii* is routinely grown using sucrose as the sole carbon source, specific genes for sucrose uptake and metabolism have not been demonstrated. Of the putative genes involved in sucrose metabolism (*scrB*, *aglA*-1, *aglA*-2 and *scrX*), none showed severe losses in fitness under any of the conditions tested (Fig. 1). While there was a slight reduction in fitness to ∼0.91 for *scrB*, this would likely not be enough to dramatically arrest growth. None of the other genes in the sucrose operon (*scrX*(Y-1)TBR) that accompany *scrB* showed any fitness defects. Since no sucrose hydrolase was identified here, it is still unknown what routes sucrose may take once inside the cell. This may also be related to potential redundancy in important pathways, which was observed previously for *A. vinelandii* for genes associated with nitrogen fixation [19]. As for sucrose transport, previous reports determined that when *A. vinelandii* was grown on melibiose (a disaccharide of galactose and glucose), the melibiose was cleaved outside the cell, leading to the accumulation of extracellular sugar monomers [29]. We do not believe this is the case for sucrose, as researchers have shown that *A. vinelandii* does not need the glucose transporter, *gluP*, to grow on sucrose, indicating that it is likely sucrose itself that is transported into the cell [5]. Our data support this conclusion, as there was no loss of fitness for the glucose transporter, *gluP* [5].

Once sucrose is converted to fructose and glucose, one gene in each of the successive pathways showed importance. The fructokinase Avin_17670, which converts fructose to fructose-6P, showed decreased fitness (∼0.68), and the other, *glk*-2, showed a similar, but lesser decrease in fitness (∼0.79), each for just the sucrose grown cultures (Fig. 1). Though *glk*-2 does not show a strong enough defect to be solely responsible for glucose conversion, this may indicate that it is the more prominent of the enzymes that could serve this purpose. Mutants deficient in *fbp* and *fba* caused severe growth defects in all but the sucrose-grown cultures. Interestingly, there were several enzymes that only showed defects in the acetate and succinate cultures (*gapB*, *pgk*, *tktA*-3 and *epd*), but not for glycerol. Since these enzymes are required for the consumption of sucrose and glycerol, as well as for gluconeogenesis, it is interesting that these enzymes only showed defects when grown on specific gluconeogenic substrates (Fig. 1).

Another pathway that could consume glucose is through the production of gluconolactone by *gcd*, but this gene had a fitness of around 1.0 for all samples. As such, the data currently indicate a possible pathway from glucose-6P to gluconate-6P in the form of *zwf* and *pgl*. While there are four *zwf* genes in *A. vinelandii*, only two were associated with this pathway in KEGG. The first, *zwf*-1, did not have any data, while the second, *zwf*-4, showed only a slight decrease in fitness (∼0.87) when grown in glycerol. Once at gluconate-6P, *A. vinelandii* uses the ED pathway when grown on sucrose [18]. The ED pathway consists of a 6-phosphogluconate dehydratase (*eda*) and a KDPG aldolase (*edd*). *A. vinelandii* has two gene copies of each, *eda*-1, *eda*-2, *edd*-1 and *edd*-2, which are co-localized in the genome. A third KDPG aldolase has also been predicted by KEGG, Avin_34340, which is not located near either of the other gene clusters. *edd*-1, *eda*-1 and Avin_34340 did not have any data, indicating that these genes may be essential to growth on sucrose. Previous reports have implicated *eda*-1 as the primary 6-phosphogluconate dehydratase due to the presence of A-rich Hfq-binding motifs, which indicate CCR control [5].

Glycerol enters central metabolism as DHAP by way of glycerol-3P [30]. *A. vinelandii* has a cluster of glycerol-related genes, *glpFKRD*. Both *glpK* and *glpD*, which are annotated as a glycerol kinase and a glycerol-3-phosphate dehydrogenase, respectively, show fitness defects only when grown on glycerol (∼0.44 and ∼0.25, respectively, Fig. 1). Of the other two genes in the glycerol cluster, *glpR* had low counts, and *glpF* showed some decreases in fitness for only glycerol-grown cells (∼0.84). In *Escherichia coli*, GlpF is thought to catalyse facilitated diffusion of glycerol across the cell membrane [31]. Another possible glycerol transporter we identified was Avin_49590, which is annotated as a citrate transporter, though this gene only showed specificity for glycerol (∼0.58).

### TCA cycle

**Fig. 2.**
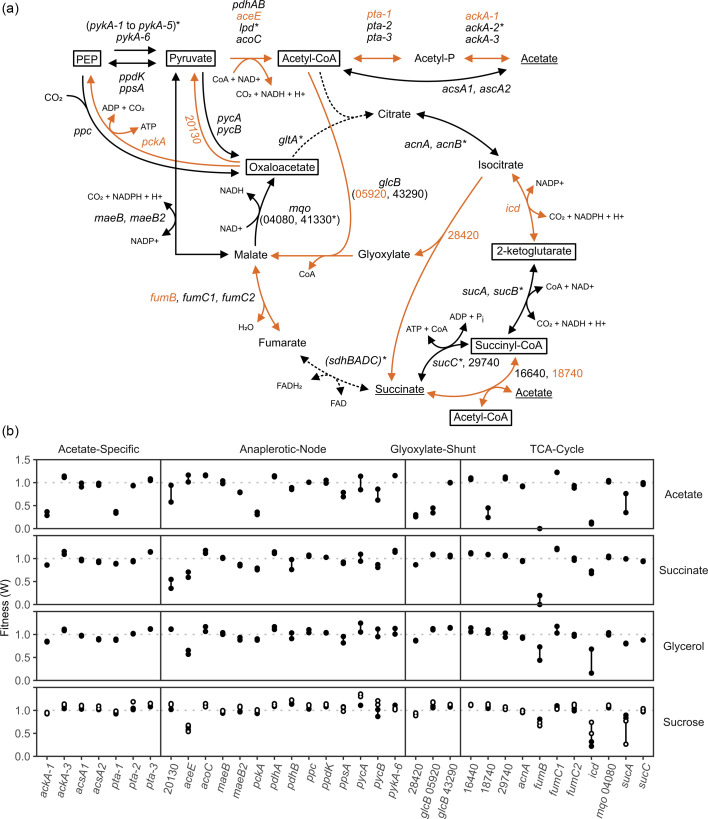
Fitness of enzymes in the TCA cycle and associated pathways in *A. vinelandii*. (**a**) TCA cycle pathways as determined by the KEGG database. The carbon sources used in this study are underlined. Dotted lines indicate that no data were obtained for that reaction. Orange lines indicate a difference was seen between carbon sources. Boxed intermediates are the essential metabolic precursors. Genes with an asterisk had low counts in the t_1_ samples. (**b**) Fitness of genes shown in panel (a). Closed circles were grown without nitrogen, while open circles were grown with nitrogen (urea). Numbers represent Avin_# designations for those genes which are not named.

In *A. vinelandii*, *ackA* and *pta* have previously been implicated in acetate metabolism [6,32]. In this pathway, *ackA* and *pta* are an acetate kinase and a phosphate acyltransferase, respectively. There are three separate clusters of these genes (*ackA*-1, *ackA*-2 and *ackA*-3 along with *pta*-1, *pta*-2 and *pta*-3) in *A. vinelandii*, but only one pair impacted acetate growth under the conditions tested, *ackA*-1 and *pta*-1 (Fig. 2). Though *ackA*-2 had no data, *pta*-2 did not show a growth defect.

There are two additional routes that could consume acetate while bringing it into the TCA cycle. The first is through direct conversion of acetate to acetyl-CoA via *ascA1* or *ascA2*. The loss of either of these enzymes did not affect growth in any of the conditions tested. The second is via a succinyl-CoA:acetate CoA-transferase, which transfers the CoA group from succinyl-CoA to a free acetate to form acetyl-CoA. Two proteins, Avin_16640 and Avin_18740, were predicted to be able to fulfil this role. Of the two, Avin_16640 had no effect, while Avin_18740 showed a severe decrease in fitness on just acetate growth. Therefore, growth on acetate seems to require a variant of the TCA cycle in which succinyl-CoA:acetate CoA-transferase replaces succinyl-CoA synthetase. This variant TCA cycle is used by acetic acid bacteria, as it provides the acetic acid resistance phenotype [33]. Acetic acid resistance typically requires other specialized enzymes that were visible with this experimental design.

Growth on acetate raises interesting questions regarding the changes to metabolism in *A. vinelandii*. Here, we identified two different entry points for acetate into the TCA cycle, via phosphorylation or CoA-transferase. Each would cause a shift in certain metabolite concentrations. One of the consequences of using a glyoxylate shunt is a loss of energy since the glyoxylate shunt skips over two steps in the TCA cycle, which create NADPH and NADH. Since *A. vinelandii* is actively fixing nitrogen under these growth conditions, it will likely have to use other pathways to produce additional NAD(P)H. Also, in some organisms, *glcB* is not an essential gene in the absence of glyoxylate production [34]. Instead, *glcB* is required for reducing the toxicity of the aldehyde glyoxylate.

In another important aspect of growing on C2 substrates, bacteria must employ a strategy that allows them to accumulate carbon by bypassing the two decarboxylating steps of the TCA cycle involving *icd* and *sucAB*. In many bacteria, this is accomplished via the glyoxylate shunt [35]. As expected, when *A. vinelandii* was grown on acetate, it also became dependent on the glyoxylate shunt. In *A. vinelandii*, the glyoxylate shunt consists of isocitrate lyase (Avin_28420) and malate synthase (*glcB*). There are two malate synthases, both named *glcB* in *A. vinelandii*, with locus tags Avin_05920 and Avin_43290. Of the two, only Avin_05920 showed a similar decrease in fitness as Avin_28420. The other malate synthase, Avin_43290, only showed a decrease when grown on galactose (∼0.73, unpublished data). In a previous report, the activities of isocitrate lyase and malate synthase in *Bradyrhizobium japonicum*, another nitrogen fixer, were tested on various carbon sources including acetate, galactose, pyruvate and glycerol [36]. While acetate had the highest activity, as expected, growth on galactose and pyruvate had intermediate activity.

Enzymes in the TCA cycle also showed changes. In the conversion of isocitrate to 2-ketoglutarate, *icd* was least important to growth on succinate (∼0.72) and most important to growth on acetate and sucrose (∼0.15 and ∼0.27, respectively). The glycerol replicates for this enzyme had too high a variance to draw strong conclusions. One of the subunits for fumarate hydratase, *fumB*, showed more importance to acetate and succinate, though we did not have high initial reads for this gene and ended with no reads in these conditions. If the change in fitness is real, it could also be due to the higher flux being shuttled through this enzyme under these conditions. Many of the core enzymes lacked data and, therefore, are likely essential to growth on sucrose, including the succinate dehydrogenase protein complex (*sdhBADC*) and citrate synthase (*gltA*). In reactions of the anapleurotic node, *pckA* was most important to creating PEP when grown on acetate (∼0.36), but there were also slight fitness defects for succinate (∼0.79) and glycerol (∼0.89). It seems that growth on succinate may prefer the use of Avin_20130, which is a hypothetical protein that is predicted to create pyruvate from oxaloacetate. Loss of this gene was most detrimental to growth on succinate (∼0.46), while less important to that on acetate (∼0.79). Finally, while most genes in the TCA cycle seemed to show differential effects for growth on either acetate or succinate, *aceE* had defects on all carbon sources but acetate. This is interesting as it is not clear why succinate would have a defect (∼0.66).

### Nitrogen-related genes

**Fig. 3.**
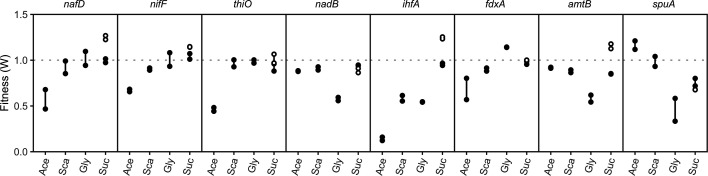
Fitness of selected nitrogen-related genes. Closed circles were grown without nitrogen, while open circles were grown with nitrogen (urea). Carbon sources are shortened as follows: Ace (acetate), Sca (succinate), Gly (glycerol) and Suc (sucrose).

In a previous report, we compared the fitness of *A. vinelandii* grown on sucrose either with or without supplemented nitrogen. In that report, we discussed genes that were in the major and minor *nif* clusters, many of which showed minimal to no fitness defects regardless of whether nitrogen was provided or not. Since *A. vinelandii* would likely not be growing on sucrose outside the laboratory, we were interested to see whether the fitness of the *nif* genes changed when grown on other carbon sources. One of these genes was a hypothetical gene in the major *nif* cluster called *nafD*. Previously, this gene did not affect diazotrophic growth on sucrose [19], but here, it did show an effect when grown on acetate (∼0.6, Fig. 3). The exact function of this protein remains to be determined, but proteins with similar domains have been found in other nitrogen fixers. Another gene, which we previously discussed, is thought to be one of the transcriptional activators for the V-nitrogenase, *vnfA3*. On sucrose, loss of this gene affected both diazotrophic and non-diazotrophic equally (fitness <0.44) and showed even more fitness impairment on all the other carbon sources. No function has yet been identified for this protein and its role in nitrogen fixation is uncertain.

Related to the transcriptional activators is the alpha subunit of integration host factor (IHF) encoded by *ihfA*. IHF is thought to bend DNA 180 degrees to bring the transcriptional activators into proximity of the RNAP [37,38]. While we did not get any insertions into the beta subunit (*ihfB*), we did not see a growth defect when grown diazotrophically or with sucrose with *ihfA* disruptions. Loss of *ihfA* showed similar growth defects for succinate (∼0.6) and glycerol (∼0.54). On acetate, the effect was much more pronounced (∼0.17). As NifA is thought to be required for diazotrophic growth on all substrates, it is interesting to consider why IHF does not influence nitrogen fixation. Typically, this enzyme is a heterodimer, though we cannot exclude the possibility that a lack of fitness may be accounted for by the creation of homodimer proteins. Deletion of the ammonium transporter *amtB* in *A. vinelandii* increases extracellular ammonium in deregulated strains [12]. Here, AmtB loss reduced fitness on glycerol (∼0.58) but showed minimal fitness loss on the other carbon sources (Fig. 3).

**Fig. 4.**
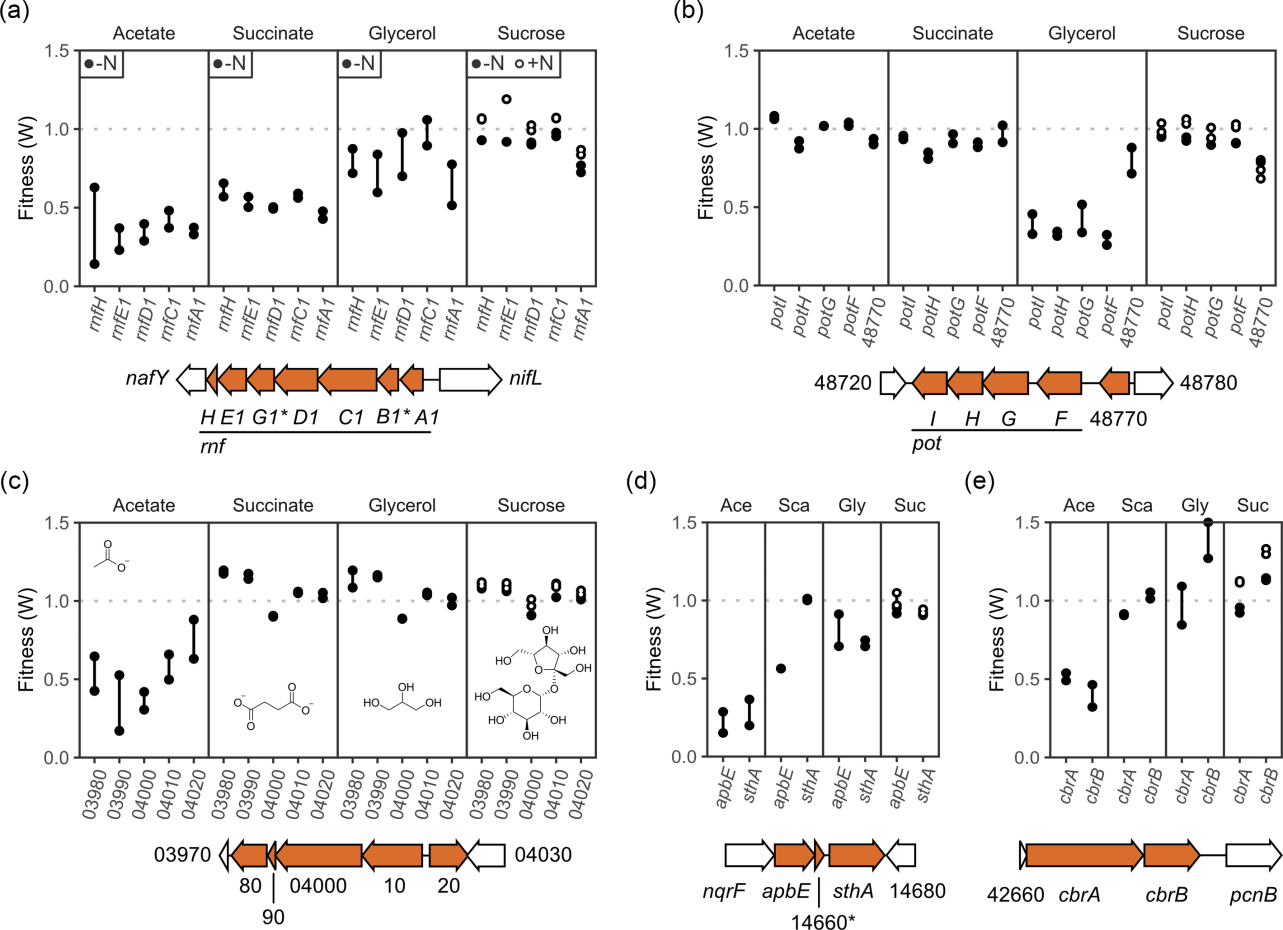
Fitness of gene groups that showed carbon-specific fitness defects. Each panel shows the fitness of genes in the cluster, while the genomic region is shown below. Plotted genes are shown in orange; flanking genes are shown in white. The figure legend is only shown in panel (a), but it is the same for all plots. In panels (d) and (e), carbon sources are shortened as follows: Ace (acetate), Sca (succinate), Gly (glycerol) and Suc (sucrose). Genes with an asterisk had low counts in the t_1_ samples.

Three different protein complexes in *A. vinelandii* have the possibility of reducing the low-potential electron donors required for nitrogenase. These three complexes are *rnf1*, *rnf2* and/or *fix* [39,40]. Of the three, the *rnf1* complex (Avin_50920–50980) showed the most significant differences between the different carbon sources (Fig. 4a). Loss of *rnf1* had the largest effect on acetate growth, with all genes showing an average fitness of less than 0.5. Succinate and glycerol growth were also affected, though to a lesser degree. Disruptions to the other Rnf2 (Avin_19220–19270) and Fix clusters showed mostly no effect on fitness between the different carbon sources and even improved growth on acetate and succinate for Rnf2. The exception to this is *fixFd*, which followed a similar trend to the Rnf1 cluster, where fitness was the lowest on acetate and then succinate, glycerol and highest on sucrose (Fig. 4). The Rnf1 complex has been demonstrated to have the greatest importance to supporting high ammonium levels in strains of *A. vinelandii* partially deregulated in BNF [41].

Related to the Rnf1 cluster is a protein named *apbE*, which flavinates the Rnf1 complex. An *apbE* deletion was equivalent to an *rnf1* deletion in *A. vinelandii* [42]. In agreement with previous reports, we found that loss of *apbE* followed the same trend as the loss of the *rnf1* cluster, where acetate was the most affected and sucrose the least (Fig. 4d). Next to *apbE* in the genome is another gene called *sthA*. SthA is annotated as a soluble pyridine nucleotide transhydrogenase and is required in growth conditions that result in excess NADPH formation. Excess NADPH is balanced by the enzyme as it converts NAD^+^ and NADPH to NADH and NADP^+^. SthA was just as important as ApbE when grown on acetate (∼0.31). Otherwise, it showed some effect on glycerol (∼0.72), but none on either succinate or sucrose. Excess NADPH is likely caused by increased flux through *icd* in the TCA cycle when grown under these conditions. Lastly, Avin_06670, a putative thioredoxin that had a similar cascading trend in fitness to that of the Rnf1 cluster. Thioredoxins are small proteins that typically have roles in redox signalling. Thioredoxin-like proteins, such as the Shethna protein in *A. vinelandii*, have roles in nitrogen fixation, as they help protect nitrogenase from oxygen damage [43].

The last of the nitrogen fixation-related genes that we looked at were those encoding the two potential electron donors, FdxA and NifF. Neither of these genes showed an effect in our previous study focused on sucrose [19], which suggested that they may simply be redundant and have an overlap of function with one another. The first, FdxA, is a ferredoxin protein that has been implicated as an electron donor to nitrogenase [44] (Fig. 3). Losing *fdxA* slightly improved growth on glycerol but decreased growth on acetate and slightly decreased growth on succinate. The other electron donor that has been implicated is called *nifF*. Unlike *fdxA*, *nifF* is a flavodoxin. Loss of *nifF* had a similar effect as *fdxA*, where there was a slight growth defect on succinate and a larger defect on acetate.

Though not directly associated with nitrogen fixation, we found a few nitrogen-associated genes that had carbon-dependent effects (Fig. 3). The first two genes, *nadB* and *spuA*, both showed the greatest fitness defects when grown on glycerol (∼0.57 and ∼0.45, respectively). The first gene, *nadB*, is annotated as an l-aspartate oxidase that would catalyse the oxidative deamination of l-aspartate to oxaloacetate, ammonia and hydrogen peroxide. This gene showed limited importance to acetate, succinate and sucrose growth (∼0.84), but was quite important to glycerol (∼0.57). l-Amino acid oxidases have been suggested to have a range of activities, including enhancing iron acquisition and catalysing keto acid production [45]. The other gene, *spuA,* is annotated in KEGG as a putative glutamine amidotransferase, though a homologue has more recently been identified as a likely *γ*-glutamyl-*γ*-aminobutyrate hydrolase in *P. aeruginosa*, where it plays a role in the catabolism of polyamines such as putrescine and spermidine [46,47]. Spermidine accumulation in the cell inhibits protein synthesis and cell viability, but this can be negated by the presence of glycerol 3-phosphate [48]. The third amino acid gene was *thiO*, a glycine oxidase. Some glycine oxidases catalyse glycine oxidation to produce glyoxylate, hydrogen peroxide and ammonia. Unlike the previous genes, *thiO* was only relevant to acetate growth (∼0.49). Since acetate is the only substrate that showed dependence on the glyoxylate shunt, this gene may relate to the glyoxylate product.

### Carbon-dependent clusters

Aside from genes in well-conserved pathways or those related to nitrogen fixation and/or ammonium, we also looked at unexpected genes that affected growth on acetate, succinate or glycerol differently than when grown on sucrose. One way we did this was by looking at clusters where consecutive genes each showed differential fitness (Fig. 4b, c and e). Two of the larger clusters each had five genes. The first contained the *pot* operon of genes, which showed importance only to growth on glycerol (Fig. 4). These genes are predicted to comprise an ABC transport system (*potFGHI*) for polyamines such as putrescine. This represents a second link between glycerol metabolism and polyamines, similar to what was found above for *spuA* [46,48]. Adjacent to the *pot* operon was a hypothetical gene, called Avin_48770. This hypothetical protein showed similar reductions in glycerol and sucrose, but no effect was observed when grown on acetate and succinate. As a means of confirming these results, we constructed the strains AZBB760 (*spuA*::tet^R^) and AZBB763 (*potHI*::tet^R^) to disrupt *spuA* and *potHI* and determine the effect these disruptions had on the ability to grow on glycerol. Loss of *spuA* resulted in the inability of *A. vinelandii* to grow on glycerol alone, but it could grow on media containing a small amount of glucose (1 g l^−1^ glucose and 19 g l^−1^ glycerol as sole carbon sources). Loss of *potHI* resulted in diminished growth on pure glycerol, but it still grew well on the glucose/glycerol mixture. Addition of a small amount of spermidine (0.2 mM) arrested growth in the *spuA*-disrupted strain, but had a negligible effect on the *potHI* disruption in the glycerol/glucose medium. Wild-type *A. vinelandii* showed a minor growth inhibition in the presence of 0.2 mM spermidine but demonstrated strong inhibition in the presence of 1.0 mM spermidine, indicating that *A. vinelandii* is not optimized for growth on certain polyamines and illustrating a strong link between polyamine metabolism and toxicity that is heightened during glycerol metabolism.

A second cluster, comprising five genes, showed importance to growth only on acetate (Avin_03980-04020). Various genes in this cluster were annotated as a HlyD family secretion protein, an RND efflux system and a fusaric acid resistance domain protein.

Several two-gene clusters showed carbon-dependent effects. One of these clusters contained two genes, which seemed related to adaptation by the strain to high salinity, Avin_10420 and *nhaA* (Avin 10430). There is also a second copy of *nhaA* (Avin_51740), which has 40% amino acid identity to Avin_10430, but which did not show any effect. Avin_10420 is a hypothetical protein, but *nhaA* is annotated as a Na^+^/H^+^ antiporter. Loss of Avin_10420 and *nhaA* caused a fitness reduction in the acetate (∼0.06 and ∼0.3) and succinate (∼0.62 and ∼0.41) cultures only. As these carbon sources were provided as sodium derivatives (sodium acetate and sodium succinate), it may be that these genes were important for adaptation to the slightly higher salinity. In *E. coli*, *nhaA* was induced by Na^+^ [49]. Another of these clusters contained *cbrA* and *cbrB* (Fig. 4e). These genes showed the greatest fitness defect on acetate, while loss of *cbrB* showed an improvement on glycerol. *cbrA* and *cbrB* comprise a two-component regulatory system in *A. vinelandii* involved in CCR [5]. Another CCR gene that showed effects was *crc* (Avin_02870), which is annotated as a catabolite repression control protein. Loss of this gene abolished the cell’s ability to grow on acetate and significantly reduced growth on glycerol. Succinate was mostly unaffected, which supports previous data suggesting that succinate is not under CCR control in *A. vinelandii* [1]. Another CCR-related gene that showed effects was *hexR*-1 (Avin_27270), a transcriptional regulator for carbohydrate utilization.

### Carbon-dependent genes

**Fig. 5.**
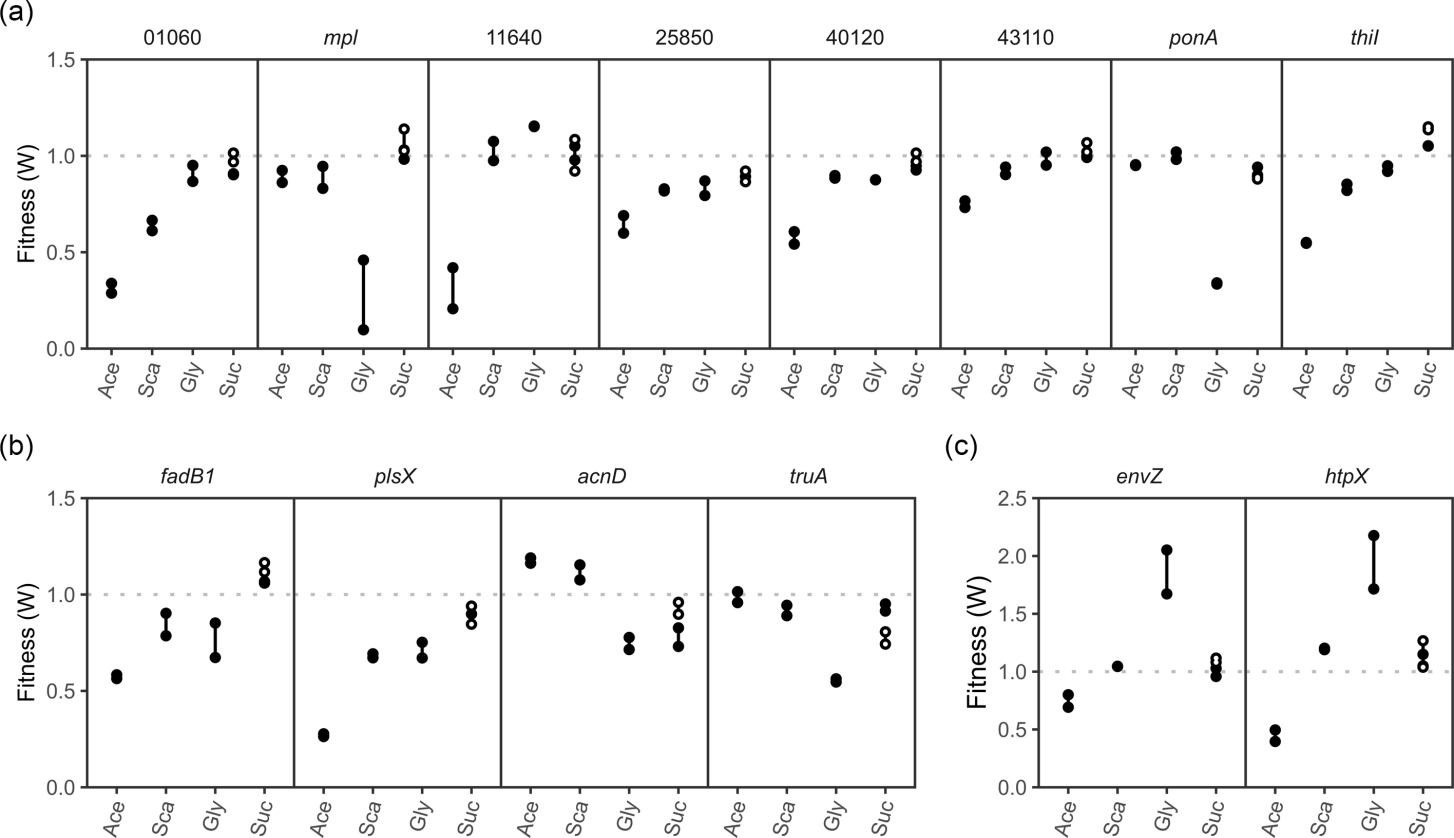
Select genes with differential fitness between carbon sources. Genes in (a) and (b) show genes predicted to encode proteins with enzymatic activity. Genes in (c) were separated due to the need for a *y*-axis that extended beyond 1.5 to 2.5. Closed circles were grown without nitrogen, while open circles were grown with nitrogen (urea). Carbon sources are shortened as follows: Ace (acetate), Sca (succinate), Gly (glycerol) and Suc (sucrose).

In addition to clusters of two or more genes, many individual genes showed carbon-dependent effects as well. Many of these genes were annotated with gene products involved in various enzymatic reactions. Avin_01060 is annotated as a carbonic anhydrase and was important for growth on acetate (∼0.34) and succinate (∼0.64). Carbonic anhydrases are important for acid acclimation and pH homeostasis in an acidic environment [50]. They convert water and carbon dioxide to carbonic acid and bicarbonate, which can act as a buffer to keep the pH from getting too low and prevent proton-motive force. Other genes also showed importance in maintaining a certain relationship with the extracellular environment. *mpl* encodes a UDP-N-acetylmuramate:L-alanyl-gamma-d-glutamyl-meso-diaminopimelate ligase and showed importance to glycerol (∼0.27). In *E. coli*, this enzyme helps to recycle cell wall peptidoglycan and assists in making new cell walls [51]. Another gene, *ponA*, encodes a penicillin-binding protein. Loss of this gene also affected only the growth of *A. vinelandii* in glycerol (∼0.33, Fig. 5). Penicillin-binding proteins also affect the cell wall by cross-linking peptidoglycan chains [52]. The importance of these two enzymes seems to indicate that the cell wall or peptidoglycan structure differs in glycerol-grown cells.

Opposite to *mpl*, two other proteins appeared to improve *A. vinelandii* growth on glycerol, while simultaneously decreasing growth on acetate. The first was *envZ*, an osmolarity-sensing histidine protein kinase [53]. Loss of *envZ* almost doubled the growth of *A. vinelandii* in glycerol (∼1.86, Fig. 5). While growth on sucrose and succinate was unaffected, acetate showed some loss of fitness (∼0.77). A heat shock protein, *htpX*, showed a very similar trend to *envZ*. Loss of this gene increased fitness when grown on glycerol (∼1.96), while decreasing fitness when grown on acetate (∼0.47). HtpX is also believed to have a role in quality control of membrane proteins [54,55].

Several genes did not lend themselves to any specific group. These genes included seven hypothetical genes that have no or limited predicted function. Avin_00730, Avin_06670 and Avin_06990 all showed severe growth defects on acetate when they were disrupted. Avin_04850 and Avin_13140 both showed growth defects when grown on glycerol. It would be interesting to see the effect that losing these genes has under more growth conditions, particularly since some of these hypotheticals are predicted to encode for small peptides (Avin_00730, 28 AA). To see if more context could be provided for these genes, we searched their predicted cellular location using pSORT. Avin_04850, Avin_06690 and Avin_45070 were predicted to be in the cytoplasmic membrane, while Avin_13140 was predicted to be a periplasmic protein. Avin_36250 had an unknown location.

### Transporters

**Fig. 6.**
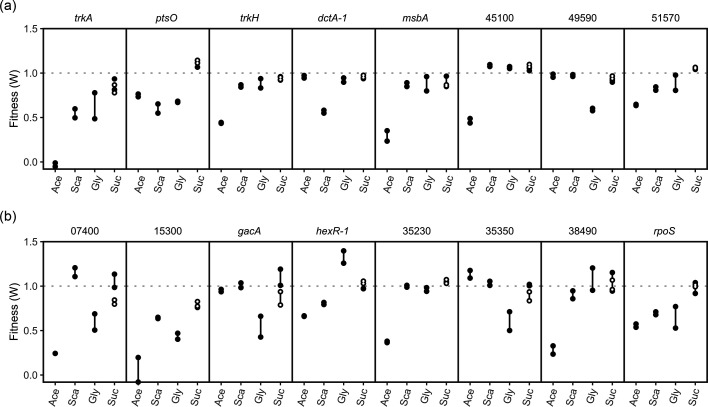
Select transport (**a**) and regulatory (**b**) genes with differential fitness between carbon sources. Closed circles were grown without nitrogen, while open circles were grown with nitrogen (urea). Carbon sources are shortened as follows: Ace (acetate), Sca (succinate), Gly (glycerol) and Suc (sucrose).

Various genes associated with cellular transport were important to growth on one or more carbon sources (Fig. 6), with most transporters only showing importance for one. One transporter showed specificity to succinate, *dctA*-1, which is annotated as a C4 carboxylic acid transporter. The exceptions to this were two genes called Avin_51570 and *ptsO*. Avin_51570 is annotated as a multidrug efflux pump RND-family outer membrane protein. Losing this gene resulted in fitness defects for all but the sucrose-grown cultures. PstO mutants had decreased growth in all but sucrose-grown cultures (fitness 0.61 to 0.77). PstO is a phosphocarrier NPr protein, which has been implicated in the nitrogen metabolic phosphotransferase system (PTS) of Gram-negative bacteria. The nitrogen PTS system is also thought to regulate potassium transport in bacteria [56]. Two additional genes were also related to potassium transport, *trkH* and Avin_45100. Different from *ptsO*, these genes only affected growth on acetate. TrkH is predicted to encode a K^+^ transporter protein, while Avin_41500 is annotated as a potassium-dependent mechanosensitive ion channel protein, and *msbA* is annotated as a lipid A exporter. Lastly, TrkA was also important but showed defects on succinate and glycerol as well.

## Conclusions

In this study, we probed how carbon metabolism differs during diazotrophic growth for acetate, succinate and glycerol versus our prior study using sucrose. As our library was isolated on sucrose and urea, we provide an examination of those genes not essential for sucrose metabolism. For each of the different carbon sources, we identified important genes specific to certain substrates that have been reported in the literature, as well as genes that have not. The challenge of Tn-seq is that it will identify which genes are important, but it will not explain why, meaning that each gene uncovered could result in an extensive set of follow-up experiments.

A great deal is known about sugar metabolism in *A. vinelandii*, as glucose and sucrose are the primary substrates used to culture this microbe in laboratory studies. There are also interesting features associated with substrate preference for organic acids versus sugars that were studied in the past, but have seen waning interest in recent years. This is more prominent for acetate but is also important for succinate, as these acids are prominent in soils and root exudates, which are common in the natural environment where *A. vinelandii* resides. Our results confirm and support many previous studies in relation to sucrose metabolism in *A. vinelandii* but identified a broad list of interesting differences for future exploration. For each of the substrates tested, we have attempted to provide context between our results and what is reported in the literature using directed approaches. Because *A. vinelandii* has an interesting preference for certain organic acids over sugars such as glucose (OAM-CCR), there are many complexities that differentiate it from other model organisms.

Here, we attempt to provide structure to this global data set, which should help define genetic differences between these growth conditions. Future studies intending to follow up on this data set include research around the Rnf1 cluster. While we predict that the Rnf1 cluster growth defects seen on acetate and succinate are related to the increased demand for ferredoxins potentially used in gluconeogenic reactions, more follow-up is needed to confirm this hypothesis. In future studies, we intend to characterize the performance of ammonium-producing strains on a variety of carbon sources. There are many questions arising from this study that will be interesting to pursue. For example, why does acetate metabolism cause an excess of NADPH and/or why does potassium transport become important? In this manner, we anticipate that these results will serve as a beneficial resource for better understanding carbon metabolism in *A. vinelandii* and contribute towards better biofertilizer strain design in the future.

## Supplementary material

10.1099/mic.0.001643 Uncited Supplementary Material 1.

## Abbreviations

B: Burk’s
BNF: biological nitrogen fixation
ED: Entner–Doudoroff
IHF: integration host factor
KEGG: Kyoto Encyclopedia of Genes and Genomes
OAM-CCR: organic acid-mediated carbon catabolite repression
PP: pentose phosphate
PTS: phosphotransferase system
Tn-seq: transposon sequencing
